# Impact of intermittent fasting on self‐regulatory behaviour and sleep in participants with insulin‐treated type 2 diabetes: A secondary analysis of the INTERFAST‐2 randomised controlled trial

**DOI:** 10.1111/dom.70469

**Published:** 2026-01-12

**Authors:** Anna Ramirez‐Obermayer, Norbert J. Tripolt, Peter N. Pferschy, Harald Kojzar, Kehkishan Azhar, Faisal Aziz, Alexander Müller, Caren Sourij, Barbara Obermayer‐Pietsch, Kristina Žukauskaitė, Angela Horvath, Vanessa Stadlbauer, Christian Vajda, Christian Fazekas, Sabrina Leal Garcia, Jolana Wagner‐Skacel, Harald Sourij

**Affiliations:** ^1^ Division of Medical Psychology, Psychosomatics and Psychotherapeutic Medicine, Department of Psychiatry, Psychosomatics and Psychotherapeutic Medicine Medical University of Graz Graz Austria; ^2^ Division of Psychiatry and Psychotherapeutic Medicine, Department of Psychiatry, Psychosomatics and Psychotherapeutic Medicine Medical University of Graz Graz Austria; ^3^ Cardiometabolic Trials Unit Medical University of Graz Graz Austria; ^4^ Division of Endocrinology and Diabetology, Department of Internal Medicine Medical University of Graz Graz Austria; ^5^ Division of Cardiology, Department of Internal Medicine Medical University of Graz Graz Austria; ^6^ Division of Gastroenterology and Hepatology, Department of Internal Medicine Medical University of Graz Graz Austria; ^7^ Center for Biomarker Research in Medicine (CBmed) Graz Austria

## 1 BACKGROUND

Intermittent fasting (IF), an eating pattern alternating fasting and eating periods, has gained scientific interest beyond weight loss.^1^ Evidence suggests IF affects physiological and psychological domains, including metabolism, stress response, and circadian rhythm regulation.^2^ Recently, research has extended to integrative outcomes like psychosomatic competence and sleep quality. Recent evidence by Ciastek et al. highlights that IF triggers a metabolic switch from glucose to fatty acid and ketone utilisation, which enhances insulin sensitivity and supports cellular homeostasis.^3^ Khalafi et al. demonstrated through meta‐analysis that IF effectively improves liver function markers (ALT, AST) and reduces liver fat in adults with metabolic disorders, regardless of the specific IF mode.^4^

Beaumont et al. utilised actigraphic recordings and found that while sleep duration may not significantly change, time‐restricted eating (TRE) leads to earlier sleep onset and fewer movements during sleep, suggesting more restorative sleep consolidation.^5^ Pavlou et al. reported that while TRE successfully reduces body weight in participants with type 2 diabetes (T2D), it does not significantly alter mood or quality of life if baseline scores are already within a healthy range.^6^

Psychosomatic competence refers to the capacity to perceive, interpret, and regulate body signals in response to internal and external stimuli and stressors. It is closely linked to self‐regulation, self‐efficacy, and associated with a lower number of bodily complaints.^7^ IF may strengthen this competence by modulating stress–response pathways, enhancing interoceptive awareness, and supporting mood regulation.^8^ Psychological and personality‐related factors play a key role in treatment adherence in chronic conditions such as type 2 diabetes (T2D), where psychotherapeutic interventions can meaningfully improve outcomes.^9^

Sleep quality, vital for health, is influenced by eating behaviour and circadian timing. IF could stabilise rhythms and improve sleep, though results remain mixed.^10^ Effective T2D management requires consistent self‐regulation across diet and exercise domains. Recent findings suggest that IF may facilitate this by inducing an “intermittent metabolic switch” from glucose to ketone utilisation, which promotes a broader metabolic recalibration.^3^ While IF and CR are comparably effective for long‐term weight loss (≥6 months), the high adherence rates often seen in TRF protocols (up to 95%) suggest it may be a sustainable self‐regulatory tool for many patients.^6^, ^11^ Furthermore, integration of exercise with IF is crucial, as it not only enhances visceral fat loss but also preserves lean body mass and cardiorespiratory fitness, addressing the physiological plateaus that often hinder long‐term adherence.^12^

This secondary analysis examines the effects of a 12‐week IF intervention on psychosomatic competence and sleep quality in insulin‐treated patients with T2D, expanding the understanding of IF's broader impact in this metabolic high‐risk group.

## 2 METHODS

### 2.1 Study

This analysis uses predefined secondary outcomes from the INTERFAST‐2 trial, an open‐label, single‐centre, randomised controlled study at Medical University of Graz, Austria. The study was approved by the local Ethics Committee (EK 30‐350 ex 17/18), registered in the German Clinical Trials Register (DRKS00018070), adhered to the Declaration of Helsinki and Good Clinical Practice, and involved written informed consent from participants.^13^

Participants in the IF group fasted three non‐consecutive days weekly (typically Monday, Wednesday, Friday), restricting intake to ~500 kcal (25% of daily needs) consumed at breakfast and/or lunch to maintain an 18‐h fast. Controls had no dietary restrictions. Both groups had equal staff contact. Psychosomatic and sleep questionnaires (in German) were completed at baseline and 12 weeks. Insulin dose was systematically reduced during the fasting days according to an insulin titration regimen published in the study protocol. Hypoglycaemia was monitored using the freestyle libre glucose monitor.^14^

**FIGURE 1:**
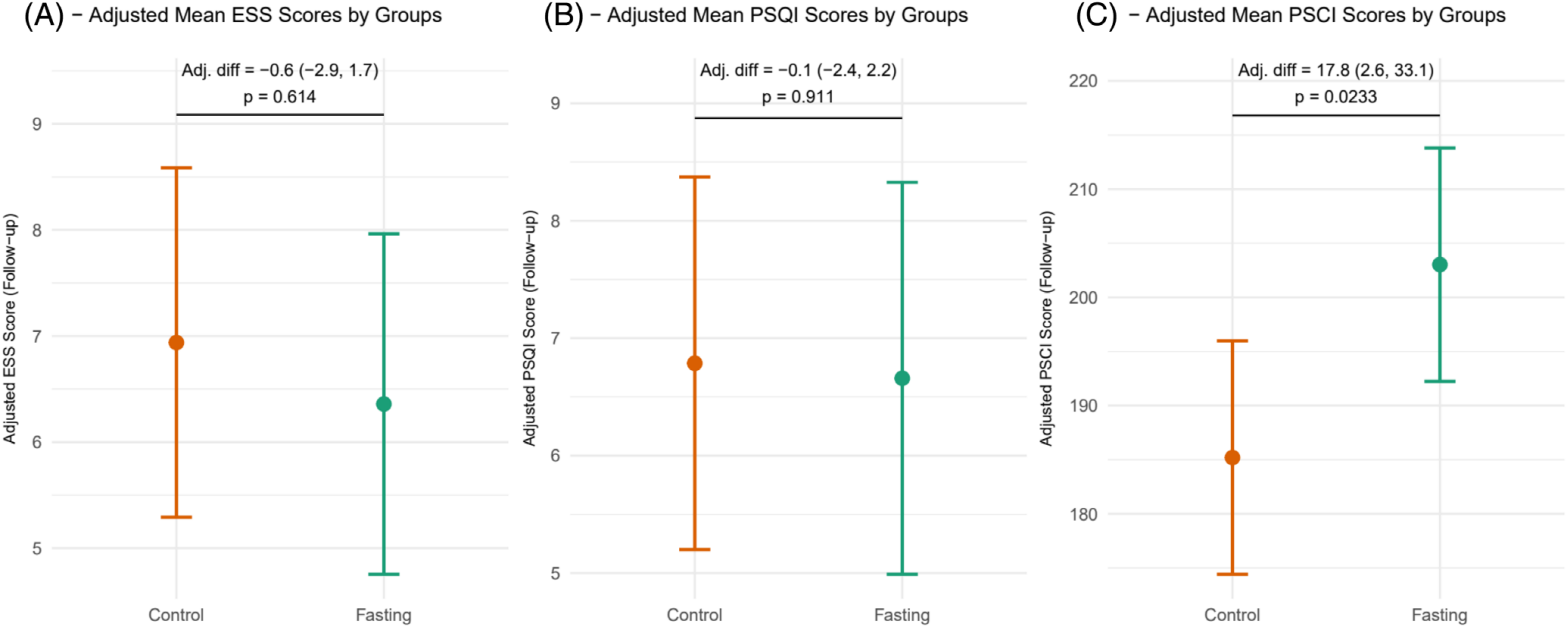
Adjusted mean (95% CI) scores at 12 weeks by intervention.

### 2.2 Epworth Sleepiness Scale

The Epworth Sleepiness Scale (ESS) consists of eight items assessing daytime sleepiness on a 0–3 scale (never to high chance of dozing).^15^

### 2.3 Pittsburgh Sleep Quality Index

The Pittsburgh Sleep Quality Index (PSQI) is a 19‐item questionnaire measuring sleep quality and disturbances over 4 weeks, covering seven components including subjective quality, latency, duration, disturbances, medication use, efficiency, and daytime dysfunction.^16^

### 2.4 Psychosomatic Competence Inventory

The Psychosomatic Competence Inventory (PSCI) is a 44‐item self‐reported questionnaire with six components: interoceptive awareness (IA), mentalisation (M), body‐related cognitive congruence (BCC), body‐related health literacy (BHL), stress experience/regulation (SER), and general self‐regulation (GSR). Higher scores indicate better bodily signal perception and stress response.^7^

### 2.5 Statistical analysis

Data were analysed using IBM SPSS Statistics (Version 29.0.0; IBM Corp., Armonk, NY) and verified in Jamovi (version 1.2.27; The Jamovi Project, Sydney, Australia) and R version 4.1.0. Repeated measures ANOVA examined effects of time (within‐subject) and group (between‐subject). Sphericity assumptions were met with two timepoints. Furthermore, linear regression was performed to compare ESS, PSQI, and PSCI scores at follow‐up between fasting and control groups. In addition, the value of each score at baseline was added as a covariate in the corresponding model to account for differences between treatment groups. The adjusted mean ± SE difference in scores at follow‐up between treatment groups was estimated and reported (Table 1).

**TABLE 1:** Linear regression comparison of ESS, PSQI, and PSCI scores between IF and control groups.

	Mean ± SEM at baseline		Adjusted mean ± SEM at follow‐up		Adjusted mean ± SEM difference (fasting − control)	*p*‐value
	Fasting	Control	Fasting	Control		
ESS	7.9 ± 0.83	6.6 ± 0.90	6.36 ± 0.79	6.94 ± 0.81	−0.58 ± 1.14	0.614
PSQI	7.3 ± 0.87	7.0 ± 0.91	6.66 ± 0.82	6.79 ± 0.78	−0.13 ± 1.14	0.911
PSCI	196.5 ± 5.4	192.3 ± 7.0	203.00 ± 5.30	185.00 ± 5.30	17.8 ± 7.50	0.023

## 3 RESULTS

Forty‐six participants (22 women, 24 men) were randomised: 22 to IF intervention and 24 to the control group. Two IF participants dropped out (1 started glucocorticoid treatment, 1 migrated to another country). Mean age was 63 ± 7 years; T2DM duration 21 ± 9 years; BMI 34.3 ± 4.5 kg/m^2^; and HbA1c 67 ± 11 mmol/mol (8.3 ± 1.1%). Mean daily insulin dose was 56 ± 27 IU. IF participants lost 4.77 ± 4.99 kg versus +0.27 ± 1.34 kg in controls (*p* < 0.001). The percentage of time below 70 mg/dL was comparable between the fasting and the control group.^13^ The use of sleep medication and antidepressants as well as other psychotropic drugs such as antipsychotics was permitted during the study. No sleep medication was regularly taken neither in the control nor in the fasting group. Regarding antidepressants 9 of the 24 patients (37.5%) in the control group and 7 of 22 patients (31.8%) in the fasting group took antidepressants (Fisher's exact test *p* = 0.76, chi‐square test *p* = 0.68) Antipsychotic medication was used by three patients in the control group (12.5%) and one patient (4.5%) in the fasting group (Fisher's exact test *p* = 0.61).

### 3.1 ESS and PSQI results

ESS scores showed no significant time effect (*F*(1,37) = 1.63, *p* = 0.210), time × group interaction (*F*(1,37) = 0.60, *p* = 0.442), or group differences (*F*(1,37) = 0.12, *p* = 0.730).

After 12 weeks, scores slightly decreased (control M = 6.90, SD = 4.54; IF M = 6.53, SD = 3.98). Repeated measures ANOVA found no significant group effect (*F*(1,38) = 0.51, *p* = 0.480), time × group interaction (*F*(1,38) = 0.002, *p* = 0.970), or change over time.

Subjective sleep quality improved in the whole cohort (F(1,35) = 10.04, *p* = 0.003), but no significant group × time interaction was found (*F*(1,35) = 0.66, *p* = 0.422).

### 3.2 PSCI results

IF participants had significantly higher PSCI scores than controls (adjusted mean difference: 17.8 ± 7.50, *p* = 0.023) after 12 weeks after adjusting for baseline scores (Figure 1). IF yielded significantly better general self‐regulation (GSR: *F*(1,34) = 15.37, *p* < 0.001), body‐related cognitive congruence (BCC: *F*(1,34) = 6.29, *p* = 0.017), and stress experience/regulation (SER: *F*(1,34) = 4.30, *p* = 0.046).

Questions showing largest differences:

1. BCC‐7: “I reach my goals even if I must give up pleasurable things.”
2. BCC‐8: “Whenever I want to change a situation (e.g., job satisfaction), several possibilities occur to me.”
3. SER‐6: “I can get myself going again without stimulants (like coffee), even when tired.”
4. SER‐7: “I can readily adjust to difficult professional and personal circumstances.”

## 4 CONCLUSION

This is the first study assessing psychosomatic competence via PSCI in insulin‐treated patients with T2D. The IF group improved more in overall PSCI and especially in general self‐regulation after 12 weeks. From the perspective of self‐efficacy and psychosomatic competence, successful participation in the IF group could be considered a 12‐week training program of body‐related self‐regulation in line with the required IF intervention. This intervention also offered other elements known to be predictors of successful behavioural change toward an active and health‐promoting lifestyle, such as planning and professional support.^17^ These aspects may all have contributed to the improvement in reported psychosomatic competence. However, further research is needed to better understand the mechanisms underlying this potential positive side effect of IF on self‐regulation skills.

ESS scores, which correlate with HbA1c and screen for sleep apnea, showed no group differences. Both groups reported improved subjective sleep quality, but average PSQI scores indicated poor sleep at baseline and after intervention. These findings align with Pavlou et al., who found no effect of time‐restricted eating on sleep quality or apnea risk in T2D over 6 months.^6^ Intermittent fasting (IF) initiates a “metabolic switch from glucose to fatty acid and ketone utilisation”^5^ providing a “metabolic recalibration” that lowers systemic inflammation.^3^ Behaviourally, this intervention serves as a “12‐week training program of body‐related self‐regulation,” strengthening the capacity to prioritise long‐term health goals over short‐term pleasure. Regarding sleep, while subjective improvements in T2D populations may remain neutral,^4^ IF was shown to improve objective sleep architecture in a recent study by “stabilising rhythms,” leading to “earlier sleep onset” and “more restorative sleep based on reduced movement.”^18^

Effective T2D management requires consistent self‐regulation in diet, exercise, and medication. Enhanced self‐regulation may help patients to achieve sustained glycaemic control and long‐term weight management.^19^ Improvements in body‐related cognitive congruence (BCC‐7, BCC‐8) indicate greater prioritisation of long‐term goals over short‐term pleasure. Better stress experience/regulation (SER‐6, SER‐7) suggests improved stress management, potentially supporting physiological stability and adherence. Active self‐regulation of bodily signals can build resilience and enhance diabetes management. This indicates that IF may have additional positive effects beyond the primary goal of weight management. The development of consistent self‐regulation in intermittent fasting (IF) could result from a dynamic interplay between internal psychological motivation and tangible behavioural correlates. The gratification associated with improved body shape as well as weight and glycaemic control acts as a powerful reinforcement that helps participants prioritise long‐term health goals. High adherence to feeding windows—which often exceeds 95% in structured time‐restricted protocols^6^—might act as a behavioural anchor that stabilises circadian rhythms and fosters the capacity to manage stress without relying on external stimulants. This behavioural stability might be further sustained by an “intermittent metabolic switch” to ketone utilisation and a significant reduction in systemic inflammation, specifically lower TNF‐α and CRP levels, which supports physiological stability and treatment adherence.^3^ Ultimately, this synergy of perceptual rewards and metabolic optimisation could strengthen the active self‐regulation of bodily signals, enhancing long‐term metabolic management.

This study extends the knowledge of IF beyond metabolic outcomes by comprehensive assessments of self‐regulatory skills and sleep in insulin‐treated people with T2D performing IF.

Limitations include unblinded glucose sensor use, possibly influencing behaviour, no dietary restrictions on non‐fasting days, and inability to blind participants due to fasting nature. Furthermore, the sample size of the trial was calculated to detect an HbA1c reduction and hence might have been underpowered for the ESS and PSQI. In addition, ESS, PSQI, and PSCI are self‐reported parameters with the associated inherent limitation. Larger, diverse cohorts and more psychological support in future research could improve PSCI validation and outcomes.

Our research suggests that IF could have additional beneficial effects beyond the previously shown weight reduction and glycaemic improvement. Further research is needed to validate these findings and explore underlying mechanisms.

## AUTHOR CONTRIBUTIONS

A.R.‐O. wrote the final manuscript. A.R.‐O., N.J.T., P.P., H.K., A.M., and C.S. contributed to the collection and interpretation of the data. K.A. and F.A. contributed to the statistical analysis. N.J.T. and H.S. contributed to acquiring ethical approval for the trial. H.S. conceived the trial. B.O.P., K.Z., A.H., V.S., C.V., C.F., S.L.G., and J.W.‐S. reviewed and contributed to the final manuscript. H.S. is the guarantor of this work and, as such, had full access to all data in the study and takes responsibility for the integrity of the data and the accuracy of the data analysis.

## FUNDING INFORMATION

This research was funded in whole or in part by the Austrian Science Fund (FWF) (https://doi.org/10.55776/PIN8074224) grants KLI 851‐B and KLI‐1076 to Harald Sourij. Clinical trial registration number is DRKS00018070, Deutsches Register Klinischer Studien (DRKS).

## CONFLICT OF INTEREST STATEMENT

The authors declare no conflicts of interest.

## Data Availability

The dataset analysed in this study is available from the corresponding author upon reasonable request.
